# National governance and excess mortality due to COVID-19 in 213 countries: a retrospective analysis and perspectives on future pandemics

**DOI:** 10.1186/s12992-023-00982-1

**Published:** 2023-10-31

**Authors:** Ricardo Eccard da Silva, Maria Rita Carvalho Garbi Novaes, Cesar de Oliveira, Dirce Bellezi Guilhem

**Affiliations:** 1 Brazilian Health Regulatory Agency - Anvisa, Setor de Indústrias, Trecho 5, Área Especial 57, Brasília-DF, 71205-050 Brazil; 2 Faculty of Health Sciences, University of Brasília – UnB, Campos Univ. Darcy Ribeiro, Asa Norte, Brasília-DF, 70910-900 Brazil; 3 Department of Epidemiology & Public Health, University College London (UCL), 1-19 Torrington Place, London, WC1E 6BT UK

**Keywords:** Covid-19, Pandemics, National governance, Excess mortality, Vaccination

## Abstract

**Background:**

National governance may have influenced the response of institutions to the Covid-19 pandemic, being a key factor in preparing for the next pandemics. The objective was to analyze the association between excess mortality due to COVID-19 (daily and cumulative per 100 thousand people) and national governance indicators in 213 countries.

**Method:**

Multiple linear regression models using secondary data from large international datasets that are in the public domain were performed. Governance indicators corresponded to six dimensions: (i) Voice and Accountability; (ii) Political Stability and Absence of Violence/Terrorism; (iii) Government Effectiveness; (iv) Regulatory Quality; (v) Rule of Law and (vi) Control of Corruption. The statistical analysis consisted of adjusting a multiple linear regression model. Excess mortality due to COVID-19 was adjusted for potential confounding factors (demographic, environmental, health, economic, and ethnic variables).

**Results:**

The indicators Control of Corruption, Government Effectiveness, Regulatory Quality and Rule of Law had a significant inverse association (p < 0.0001) with the estimated excess mortality in 2020, 2021 and 2022. Furthermore, the governance indicators had a direct significant association (p < 0.0001) with the vaccination variables (People_fully_vaccinated; Delivered population; The total number of vaccination doses administered per 100 people at the country level), except for the variables Vaccination policies and Administration of first dose, which were inversely associated. In countries with better governance, COVID-19 vaccination was initiated earlier.

**Conclusion:**

Better national governance indicators were associated with lower excess mortality due to COVID-19 and faster administration of the first dose of the COVID-19 vaccine.

**Supplementary Information:**

The online version contains supplementary material available at 10.1186/s12992-023-00982-1.

## Introduction

The factors that contributed to their success were the implementation of fast and timely responses, high testing capacity, efficient contact tracing, strict social isolation measures, equitable distribution of vaccines, and prioritization of vulnerable groups. Additionally, government leadership capacity, public trust, clear communication of risks and strategies based on scientific evidence were essential in combating the COVID-19 pandemic [1]. In addition to the factors early action, rapid testing, complete contact tracing and quarantine, other factors such as social and economic protection of individuals, especially the most vulnerable, may have contributed in the best responses [2, 3].

The effectiveness of government measures and the capacity of public institutions were the determining factors in controlling and eliminating the virus’s spread. Governance played a crucial role in countries’ response to the COVID-19 pandemic, and it will be a key factor in preparing for future pandemics. The lessons learned from this pandemic showed, mainly, the need to control of corruption and building public trust in institutions [4].

According to the World Bank, Governance refers to the traditions and institutions that govern authority in a country. This includes the process of selecting, monitoring, and replacing governments, the capacity of the government to develop and implement effective policies, and the mutual respect between citizens and the state for economic and social institutions. The dimensions of national Governance include government effectiveness, regulatory quality, rule of law, control of corruption, political stability, non-violence, voice, and accountability [5].

Restrictive measures implemented to curb the spread of COVID-19 were more effective in countries with good governance and lower levels of corruption. The dimensions of governance, namely government effectiveness, regulatory quality, rule of law, and control of corruption, had a significant impact on the increased availability of medical supplies and personal protective equipment [6].

The Rule of Law plays a crucial role in responding to public health emergencies like the pandemic as it requires that the measures taken by governments are based on legality, legitimacy, transparency, and fairness. These measures should also have participatory and inclusive approaches that guarantee fundamental rights [7–9].

Governmental inefficiency and corruption have resulted in a slow implementation of recommended health measures. The restriction of movement alone has not been sufficient to curb the spread of the pandemic [10]. Studies have indicated that mortality rates for COVID-19 are not necessarily tied to strict government measures. In addition, good governance is not necessarily correlated with strict enforcement [11]. Factors such as population density, a higher proportion of vulnerable occupations, and increased international travel have contributed to the spread of the virus [10].

Trust in local and national public health institutions has been linked to higher compliance with non-pharmaceutical interventions such as mask-wearing [12]. Additionally, higher levels of trust in government and interpersonal trust, and lower levels of government corruption, have been associated with reduced rates of infection by SARS-CoV-2.

There is a significant variation in the rate of SARS-CoV-2 infections across different countries, indicating the need for further research into other possible variables, such as community involvement and risk communication [13]. To prepare for future pandemics, it is essential to study the factors that contributed to the excess mortality caused by COVID-19. Therefore, the objective of this study was to analyze the relationship between COVID-19 outcomes and national governance indicators in 213 countries.

## Methods

### Study design

This study has analysed large international secondary datasets that are in the public domain. Data from 213 countries were used in the analysis. The complete list of 213 countries is presented in supplementary file 1. Governance indicators data were from 2020. For the outcome referring to the daily and cumulative excess deaths per 100 thousand, three periods were used: (i) January 1 to December 7, 2020; (ii) January 1, 2020 to December 6, 2021 and (iii) January 1, 2020 to December 5, 2022. The choice of 2020 is justified by the declaration of a public health emergency by the World Health Organization. Considering the development and use of COVID-19 vaccine in 2021 and 2022, these years were included.

### Data sources

The databases used were: (i) World Governance Indicators – World Bank; (ii) GovData360 – World Bank; (iii) Data Futures Platform (United Nations Development Programme); (iv) The Economist; (v) Oxford COVID-19 Government Response Tracker – University of Oxford; (vi) Our World in Data – University of Oxford and Global Change Data Lab and (vii) Vaccine Equity Dashboard Data (United Nations Development Programme).

World Governance Indicators [5] are a publicly accessible research dataset, managed by the World Bank, which shows world development indicators, including those related to country governance.

GovData360 [14] is an initiative of the World Bank’s Governance Global Practice. This database contains more than 4,700 governance-related indicators on state capacity, efficiency, openness, inclusiveness, accountability, integrity and trust in government. The site gathers information from 35 data sources, including other World Bank sources.

The Data Futures Platform brings together data from the United Nations system [15] and partners to advance integrated development solutions in support of the 2030 Agenda. The platform includes raw data sets, simulators and actionable insights, allowing users to both run their own estimation and access relevant analyses to inform policies and programs.

The Economist [16] has built a machine-learning model which estimates excess deaths during the pandemic in 223 countries.

The Oxford COVID-19 Government Response Tracker [17] systematically collects and aggregates data on government responses to the pandemic. The data are from more than 180 countries and correspond to 23 indicators.

Our World in Data [18] is a collaborative effort between researchers at the University of Oxford and a non-profit organization, Global Change Data Lab. This database concentrates indicators of poverty, disease, hunger, climate change, war, existential risks and inequality, as well as indicators of the COVID-19 pandemic.

Vaccine Equity Dashboard Data [19] is a joint initiative from United Nations, World Health Organization and the University of Oxford, and provides information on the distribution and vaccine equity in countries.

### Selection of variables

#### Governance

It corresponds to six dimensions: (i) Voice and Accountability; (ii) Political Stability and Absence of Violence/Terrorism; (iii) Government Effectiveness; (iv) Regulatory Quality; (v) Rule of Law and (vi) Control of Corruption. The definitions, database and year of the variables are presented in supplementary file 2.

#### Infection rates and daily and cumulative excess deaths per 100 thousand

Cumulative infection rate and infection-fatality ratio from 2021 were used in a published study [13] which assessed the association between governance, health, economic indicators and COVID-19 outcomes. Excess mortality estimates how many people died during the COVID-19 pandemic relative to the expected number of deaths under normal conditions [20]. Excess mortality corresponds to a more comprehensive assessment of the impact of the pandemic than to the number of confirmed deaths due to Covid-19, as it also considers misdiagnosed or misreported deaths and mortality resulting from overburdened health services or exacerbated poverty [16, 21].

### Confounding factors

These factors have been referred to as covariates. They are demographic, environmental, health, economic, and ethnic [13, 22, 23]. The confounding factors considered were:

- Population aged 65 and older (% of total population) [24].
- Estimated population (%) at < 5 m elevation (Altitude) [24].
- Population density (people per sq. km of land area) [24].
- GDP per capita (current US$) [24].
- Estimated population (%) at < 500 m elevation (Altitude) [25].
- Ambient particulate matter pollution (micrograms per cubic meter) [26].
- Age-Standardized Smoking Prevalence (15 + years) [26].
- High body mass index (BMI) [26].
- Asthma Prevalence [26].
- Total Cancer Prevalence [26].
- Chronic obstructive pulmonary disease Prevalence [26].
- Diabetes mellitus Prevalence [26].
- Cardiovascular diseases Prevalence [26].
- Tuberculosis Prevalence [26].
- Alzheimer’s disease and other dementias Prevalence [26].
- Ethnic Fractionalization [27].

The likelihood of evolving to the severe form of COVID-19 was assessed as a possible confounding factor in relation to excess mortality due to COVID-19 [28].

Factors related to vaccination (People_fully_vaccinated; Delivered population; Vaccination policy; Administration of the first dose in the country; The total number of vaccination doses administered per 100 people at the country level) were also assessed as possible confounders of this excess mortality due to COVID-19, and infection rates adjusted for one thousand people and infection-fatality ratio adjusted for one thousand infections [29–32].

### Statistical analysis

Statistical analysis consisted of adjusting a multiple linear regression model to analyze the association between the daily estimated cumulative excess deaths per 100 thousand inhabitants, in 2020, 2021 and 2022, and governance variables. Excess mortality was adjusted for possible confounding factors: demographic, environmental, ethnic, economic, health, and vaccination covariates. Vaccination covariates were considered for the years 2021 and 2022. The statistical model also assessed the association between COVID-19 outcomes related to infection rates adjusted for one thousand people and infection-fatality ratio adjusted for one thousand infections (2021), adjusted for possible confounders (vaccination covariates) and the governance variables. Also, the model was used to assess the association of governance indicators with vaccination variables. In the case of the vaccination policy variable, it was dichotomized into two values: 0 (zero), if the Vaccination policies were from the year 2021; and 01 (one), if the Vaccination policies were from Jan to Jun 2022. The variable Administration of the first dose in the country was dichotomized into two values: 0 (zero) if the Administration of the first dose in the country occurred from Jul to Dec 2020; and 01 (one) if the administration took place from Jan to Oct 2021.

The daily estimated cumulative excess deaths per 100 thousand inhabitants, in 2020, 2021 and 2022 and infection rates and infection-fatality ratio adjusted for one thousand infections were considered the dependent variables. Those of governance were considered as independent variables of interest, and as covariates, which were considered as possible confounding factors.

The analysis occurred in two steps: (i) initially, to determine whether the demographic, environmental, ethnic, economic and health covariates were associated with the daily estimated cumulative excess deaths per 100 thousand inhabitants, in 2020, 2021 and 2022, a model of stepwise linear regression was adjusted and those with p-value < 0.05 remained in the model. The model was also used to determine whether the vaccination covariates were associated with infection rates adjusted for one thousand people and infection-fatality ratio adjusted for one thousand infections; (ii) in the second stage, those related to governance were included in order to verify which ones would be significantly associated with the dependent variable, after adjusting for possible confounders. However, it was observed that the governance indicators showed a strong correlation with each other and, therefore, a strong multicollinearity. A principal component analysis was used on the governance variables in order to generate factors orthogonal to each other to avoid the effect of multicollinearity [33]. This analysis resulted in three components: PRIN1, PRIN2, PRIN3. Finally, a model with the three main components and significant confounders was adjusted. Assumptions of normality and homoscedasticity were verified through the analysis of residual graphs and probabilistic normal, as well as the identification of possible outliers and leverage points. The analyzes were conducted using the Statistical Analysis System 9.4 [34].

The methodological flow is presented in supplementary file 3.

## Results

### Excess mortality in 2020 and governance variables

After considering demographic, environmental, ethnic, economic, and health factors, only three covariates remained in the model with a p-value < 0.05. These covariates were the percentage of the population aged 65 and older, the estimated population percentage at an altitude lower than 500 m, and the rate of high BMI. The percentage of the population aged 65 and older and the rate of high BMI were significantly and directly correlated with each other (p < 0.0001 for both). On the other hand, the estimated population percentage at an altitude lower than 500 m was significantly inversely correlated with the daily estimated cumulative excess deaths per 100 thousand inhabitants in 2020 (p = 0.0003).

After including these three covariates, only the first principal component (PRIN1) was significant (p < 0.0001). This means that the dimension to which the variable PRIN1 refers was inversely related to the daily estimated cumulative excess deaths per 100 thousand inhabitants in 2020. However, both the PRIN2 and PRIN3 components were not associated with the daily estimated cumulative excess deaths per 100 thousand inhabitants in 2020 (p-value of 0.5470 and 0.3507, respectively) (Table 1). Together, these three components explain 96.75% of the total variability. The adjusted model had an R2 value of 0.5460.

**Table 1:** Multiple linear regression analysis of governance variables, adjusted by demographic, environmental, ethnic, economic and health covariates on the daily estimated cumulative excess deaths per 100 thousand inhabitants, in 2020 (n = 143)

Variables	Parameter Estimate	Standard Error	T-statistics	p-value	Standardized Parameter Estimate
Intercept	-53,37505	14,28006	-3,74	0.0003	0
Population aged 65 and older (% of total population)	9,15335	0,88996	10,29	< 0.0001	0,94024
Estimated population (%) at < 500 m elevation (Altitude)	-0,46399	0,12538	-3,70	0.0003	-0,23083
BMI (rate)	2,31947	0,40501	5,73	< 0.0001	0,38717
^1^Prin1	-22,09834	2,53009	-8,73	< 0.0001	-0,76137
^2^Prin2	-4,16107	6,89196	-0,60	0.5470	-0,03768
^3^Prin3	7,11961	7,60270	0,94	0.3507	0,05694

^1^Control of Corruption, Government Effectiveness, Regulatory Quality and Rule of Law

^2^Voice and Accountability

^3^Political Stability and Absence of Violence/Terrorism

### Excess mortality in 2021 and governance variables

After accounting for various factors such as demographics, environment, ethnicity, economy, and health, the following covariates were found to be significant: the percentage of the population aged 65 and older, the estimated percentage of the population at an altitude of less than 500 m, and a high BMI rate (with a p-value of less than 0.05). However, after including the three main components, only the first one (control of corruption, government effectiveness, regulatory quality, and rule of law) was found to be statistically significant (with a p-value of less than 0.0001). Lastly, vaccination-related factors were not found to be associated with the excess mortality outcome.

The percentage of the population aged 65 and older and the rate of high BMI were found to be significantly and directly correlated (p < 0.0001 for both). On the other hand, the estimated population percentage living at an altitude of less than 500 m was inversely correlated (p = 0.0015) with the daily estimated cumulative excess deaths per 100 thousand inhabitants in 2021. In terms of governance variables, only the first component showed a significant inverse correlation (p < 0.0001) with the daily estimated cumulative excess deaths per 100 thousand inhabitants in 2021. The PRIN1 was inversely related to the daily estimated cumulative excess deaths per 100 thousand inhabitants in 2021, while PRIN2 and PRIN3 did not show any correlation with the daily estimated cumulative excess deaths per 100 thousand inhabitants in 2021 (p = 0.8823 and p = 0.3658, respectively) (Table 2). These three components together explain 96.87% of the total variability. The adjusted model provided an R2 value of 0.5033.

**Table 2:** Multiple linear regression analysis of governance variables, adjusted by demographic, environmental, ethnic, economic and health covariates on the daily estimated cumulative excess deaths per 100 thousand inhabitants, in 2021 (n = 132)

Variables	Parameter Estimate	Standard Error	T-statistics	p-value	95% CI Limits
Intercept	-54,71	15,61	-3,50	0.0006	-85,61; -23,80
Population aged 65 and older (% of total population)	8,99	1,03	8,77	< 0.0001	6,96; 11,02
Estimated population (%) at < 500 m elevation (Altitude)	-0,46	0,14	-3,25	0.0015	-0,74; -0,18
BMI (rate)	2,27	0,46	4,90	< 0.0001	1,35; 3,18
^4^Prin1	-21,84	2,84	-7,70	< 0.0001	-27,45; -16,23
^5^Prin2	1,12	7,58	0,15	0.8823	-13,88; 16,13
^6^Prin3	-7,79	8,59	-0,91	0.3659	-24,79; 9,20

^4^Control of Corruption, Government Effectiveness, Regulatory Quality and Rule of Law

^5^Political Stability and Absence of Violence/Terrorism

^6^Voice and Accountability

### Excess mortality in 2022 and governance variables

After adjusting for various social, demographic, economic, and health factors, some covariates remained significant in the analysis. These covariates include the percentage of the population aged 65 and above, the estimated population living at an elevation of less than 500 m, the rate of high BMI, and the total prevalence of cancer (with a significance level of p < 0.05). After including three principal components, the analysis showed that only PRIN1 and PRIN2 were significant (with a significance level of p < 0.0001 and p = 0.0469, respectively). Vaccination-related factors were not associated with the excess mortality outcome. The covariates, population aged 65 and above and high BMI rate, were significantly and directly correlated (with a significance level of p < 0.0001 for both). On the other hand, the estimated population living at an elevation of less than 500 m and total cancer prevalence were inversely correlated (with a significance level of p = 0.0022 and p < 0.0001, respectively) with the daily estimated cumulative excess deaths per 100 thousand inhabitants in 2022. Regarding the variables related to governance, PRIN1 was inversely and significantly correlated (with a significance level of p < 0.0001), while PRIN2 was directly and significantly correlated (with a significance level of p = 0.0469). PRIN3, however, was not correlated with the daily estimated cumulative excess deaths per 100 thousand inhabitants in 2022 (with a significance level of p = 0.6404) (Table 3). These three components explain 96.87% of the total variability. The adjusted model provided an R2 value of 0.6177.

**Table 3:** Multiple linear regression analysis of governance variables, adjusted by demographic, environmental, ethnic, economic and health covariates on the daily estimated cumulative excess deaths per 100 thousand inhabitants, in 2022 (n = 131)

Variables	Parameter Estimate	Standard Error	T-statistics	p-value	95% CI Limits
Intercept	-109,96	36,73	-3,08	0.0026	-180,68; -39,24
Population aged 65 and older (% of total population)	35,26	3,29	10,72	< 0.0001	28,74; 41,77
Estimated population (%) at < 500 m elevation (Altitude)	-1,02	0,33	-3,12	0.0022	-1,66; -0,37
BMI (rate)	6,65	1,05	6,34	< 0.0001	4,58; 8,73
Total Prevalence of Cancer	-0,09	0,02	-5,12	< 0.0001	-0,13; -0,06
^7^Prin1	-39,81	7,09	-5,62	< 0.0001	-53,83; -25,78
^8^Prin2	34,57	17,22	2,01	0.0469	0,48; 68,66
^9^Prin3	9,15	19,53	0,47	0.6404	-29,52; 47,82

^7^Control of Corruption, Government Effectiveness, Regulatory Quality and Rule of Law

^8^Political Stability and Absence of Violence/Terrorism

^9^Voice and Accountability

### Infection rates adjusted per thousand people and infection-fatality ratio adjusted per thousand infections (2021) and the governance variables

After adjusting for vaccination covariates, the following variables remained in the model: vaccination policies (Jan to Jun 21; Jul to Dec 21 and Jan to Jun 22) and administration of the first dose in the country (Jul to Dec 20 and Jan to Oct 21) (p < 0.05). After the inclusion of the three main components, only PRIN1 and PRIN2 proved to be significant (p = 0.0032 and p = 0.0333, respectively). The covariate administration of first dose in the country became no longer significant and was removed from the model. The covariate infection-fatality ratio adjusted for one thousand infections was not associated with governance indicators.

The covariate vaccination policies in the Jan to Jun 2022 category were found to be significant (p = 0.0086) and presented an estimate of -202.89 when compared to the Jan to Jun 2021 category. This means that the infection rate per 1000 inhabitants was, on average, 202.89 lower in countries that achieved universal availability in a later period (Jan to Jun 2022) compared to countries that achieved it earlier (Jan to Jun 2021). That is, on average 202,890 fewer people were infected with Covid-19 in countries that achieved universal availability in a later period “Jan to Jun 2022” compared to countries that achieved universal availability in an earlier period “Jan to Jun 2021”. Countries that made vaccinations available to the entire population later had a lower rate of accumulated infections compared to countries that made them available earlier.

Regarding the variables related to governance, PRIN1 was found to correlate inversely and significantly (p < 0.0001), while PRIN2 was found to correlate directly and significantly (p = 0.0333). As for PRIN3, it was not found to be correlated with the infection rate in 2021 (p = 0.5903) (Table 4). These three components explain 97.12% of the total variability. The adjusted model provided an R2 value of 0.1087.

**Table 4:** Multiple linear regression analysis of governance variables, adjusted by socio, demographic, economic and health covariates on the adjusted infection rate per thousand people, in 2021 (n = 131)

Variables	Parameter Estimate	Standard Error	T-statistics	p-value	95% CI Limits
Intercept	465,31	38,92	11,96	< 0.0001	388,36; 542,26
Vaccination policies (Jul to Dec 2021 x Jan a Jun 2021)	-34,23	46,86	-0,73	0.4663	-126,89; 58,43
Vaccination policies (Jan to Jun 2022 x Jan to Jun 2021)	-202,89	76,06	-2,67	0.0086	-353,29; -52,51
^10^Prin1	-28,82	9,61	-3,00	0.0032	-47,84; -9,80
^11^Prin2	77,70	36,14	2,15	0.0333	6,23; 149,16
^12^Prin3	23,28	43,14	0,54	0.5903	-62,02; 108,60

^10^Control of Corruption, Government Effectiveness, Regulatory Quality and Rule of Law

^11^Voice and Accountability

^12^Political Stability and Absence of Violence/Terrorism

### Vaccination variables and governance variables

The study found that there is a significant and direct relationship between governance indicators and vaccination rates. In other words, countries with better governance indicators had a higher number of people fully vaccinated. However, the data also showed that both vaccination policies and administration of the first dose had a significant and inverse relationship with governance indicators. This means that countries that implemented vaccination policies earlier or administered the first dose later had lower governance indicators. The results are summarized in Table 5.

**Table 5:** Pearson Correlation Coefficients and Biserial Correlation of governance indicators and vaccination variables

Governance Indicators	Vaccination Variable	N	Correlation Estimate	95% CI Limits		p Value for H0:Rho = 0
Control_Corruption	Total_number__vaccination_doses	196	0.61332	0.517665	0.693832	< 0.0001
Government_Effectiveness	Total_number__vaccination_doses	196	0.69941	0.620036	0.764612	< 0.0001
Political_Stability	Total_number__vaccination_doses	200	0.56995	0.468231	0.656755	< 0.0001
Regulatory_Quality	Total_number__vaccination_doses	196	0.61810	0.523273	0.697800	< 0.0001
Rule_Law	Total_number__vaccination_doses	196	0.62836	0.535353	0.706310	< 0.0001
Voice_Accountability	Total_number__vaccination_doses	198	0.40167	0.277788	0.512412	< 0.0001
Control_Corruption	People_fully_vaccinated	196	0.59922	0.501159	0.682092	< 0.0001
Government_Effectiveness	People_fully_vaccinated	196	0.68324	0.600600	0.751437	< 0.0001
Political_Stability	People_fully_vaccinated	200	0.57946	0.479249	0.664759	< 0.0001
Regulatory_Quality	People_fully_vaccinated	196	0.61960	0.525036	0.699045	< 0.0001
Rule_Law	People_fully_vaccinated	196	0.61513	0.519785	0.695333	< 0.0001
Voice_Accountability	People_fully_vaccinated	198	0.39736	0.273049	0.508620	< 0.0001
Control_Corruption	Delivered_population	191	0.64786	0.557122	0.723304	< 0.0001
Government_Effectiveness	Delivered_population	191	0.70387	0.624279	0.769000	< 0.0001
Political_Stability	Delivered_population	192	0.62076	0.525328	0.700766	< 0.0001
Regulatory_Quality	Delivered_population	191	0.63609	0.543163	0.713620	< 0.0001
Rule_Law	Delivered_population	191	0.65853	0.569832	0.732066	< 0.0001
Voice_Accountability	Delivered_population	191	0.47823	0.360742	0.580774	< 0.0001
Control_Corruption	Vaccination_policies (Jan Jun 22)	175	-0.30962	-0.437852	-0.169041	< 0.0001
Government_Effectiveness	Vaccination_policies (Jan to Jun 22)	175	-0.36047	-0.482987	-0.224111	< 0.0001
Political_Stability	Vaccination_policies (Jan to Jun 22)	177	-0.27808	-0.408812	-0.136164	0.0002
Regulatory_Quality	Vaccination_policies (Jan to Jun 22)	175	-0.29312	-0.423063	-0.151353	< 0.0001
Rule_Law	Vaccination_policies (Jan to Jun 22)	175	-0.33082	-0.456748	-0.191894	< 0.0001
Voice_Accountability	Vaccination_policies (Jan to Jun 22)	175	-0.28133	-0.412458	-0.138776	0.0001
Control_Corruption	Administration_first_dose (Jan Out 21)	197	-0.45983	-0.563405	-0.342015	< 0.0001
Government_Effectiveness	Administration_first_dose (Jan Out 21)	197	-0.47612	-0.577480	-0.360307	< 0.0001
Political_Stability	Administration_first_dose (Jan Out 21)	201	-0.31152	-0.431319	-0.180925	< 0.0001
Regulatory_Quality	Administration_first_dose (Jan Out 21)	197	-0.50698	-0.603967	-0.395189	< 0.0001
Rule_Law	Administration_first_dose (Jan Out 21)	197	-0.50786	-0.604725	-0.396196	< 0.0001
Voice_Accountability	Administration_first_dose (Jan Out 21)	197	-0.39257	-0.504669	-0.267452	< 0.0001

## Discussion

Based on the analysis of the years 2020 and 2021, it was found that among the three main components examined, only the “Control of Corruption, Government Effectiveness, Regulatory Quality and Rule of Law” component had a significant impact (p < 0.0001). In other words, the component represented by the variable PRIN1 was found to be inversely related to the daily estimated cumulative excess deaths per 100 thousand inhabitants in the year 2020. On the other hand, the “Voice and Accountability” component and the “Political Stability and Absence of Violence/Terrorism” component did not show any correlation with the daily estimated cumulative excess deaths per 100 thousand inhabitants. These findings are consistent with previous studies that have shown that countries with good governance, including trust in government, have lower excess mortality rates due to COVID-19 [35–37] and lower mortality rates and infection-fatality ratio and infection attack rates of COVID-19 [38–40].

Few studies have examined the link between governance indicators and excess mortality caused by COVID-19. This study has a strength in that it utilized the more robust outcome of excess mortality caused by COVID-19. Comparing the number of deaths due to COVID-19 may not be the most suitable method for comparing different countries [41]. It is challenging to discuss the results of studies that have examined different outcomes of COVID-19.

An ecological study revealed a link between worse governance indicators and lower COVID-19 mortality among 54 African countries. However, the authors of the study believed that the high number of patients aged 65 or older and the use of the total number of deaths instead of weekly averages may have affected the results [42]. Another research found that the World Bank governance indicators were not related to the number of cases and deaths from COVID-19 [43]. Although these studies focused on the mortality rate due to COVID-19, the analysis of the association between governance and COVID-19 outcomes still seems debatable and requires further research.

A previous study that evaluated three dimensions of the governance indicator, namely Government Effectiveness, Regulatory Quality, and Rule of Law found that countries with high levels of these dimensions had better performances during the COVID-19 pandemic, such as lower mortality rates [44]. This suggests that the governance indicator, even if it lacks all six dimensions, still played a significant role in the performance of countries during the pandemic.

The rule of law has, among its dimensions, legality, proportionality, transparency and the participatory process in the drafting and implementation of laws and regulations. Government communication in a transparent manner helps individuals to understand the reason for implementing the government measures adopted [45]. Therefore, in a country with a strengthened rule of law, this understanding may have influenced citizens’ perception that their rights and freedoms were not being violated [46]. The rule of law provides legitimacy to the government’s restrictive measures. The limitations of some rights must be proportional. That is, the least restrictive measures should be implemented to deal with the situation. Adherence to rule of law principles, when adopting emergency measures, is expected to strengthen public trust in the institutions. Therefore, compliance with measures and effectiveness of actions can be reinforced [47].

It is interesting to note that the results of the current research indicate that in the years 2020 and 2021, the components “Voice and Accountability” and “Political Stability and Absence of Violence/Terrorism” did not have any correlation with the excess mortality rate caused by COVID-19. However, in 2022, the “Political Stability and Absence of Violence/Terrorism” component was directly and significantly associated with the excess mortality rate due to COVID-19. It is important to understand that the World Bank’s interpretation of the “Political Stability and Absence of Violence/Terrorism” and “Voice and Accountability” indicators [5] is related to the citizens’ engagement in the government’s selection process, freedom of expression, and the media’s ability to function freely.

A study conducted in 203 countries aimed to identify the possible predictors of mortality due to COVID-19 found that the “Voice and Accountability” indicator was a positive predictor. The researchers argued that this could be due to better governance leading to a better reporting system for deaths [48]. However, the results of the present research contradict this finding. The lack of transparency in data reporting may have affected the reporting of cases and deaths. This may have been a factor that influenced the present study’s results, which found no significant association between excess mortality and the “Voice and Accountability” indicator.

Another study analyzed data from 185 countries to investigate the relationship between pandemic spread namely the COVID-19 positive rate and the COVID-19 growth rate i.e., the quantity of testing being done in a country relative to the magnitude of the outbreak, and all six dimensions of governance also analysed in the present study, including the “Voice and Accountability” indicator and the “Political Stability and Absence of Violence/Terrorism” indicator. The study found that these indicators had a smaller influence on COVID-19 positive rates compared to the other dimensions of governance. The researchers suggested that the “Voice and Accountability” and “Political Stability and Absence of Violence/Terrorism” indicators are more specific and may not strongly influence the quality of governance as the “Government Effectiveness” indicator does [49].

A recent study analyzing data from 226 countries found that the effectiveness of non-pharmaceutical interventions to mitigate the spread of COVID-19 was negatively impacted by two factors - “Voice and Accountability” and “Political Stability and Absence of Violence/Terrorism”. Countries that value freedom of expression and free media, which are some aspects of “Voice and Accountability”, may have faced more challenges in implementing non-pharmaceutical interventions, especially when such interventions were imposed [6].

By 2022, some countries had made more progress in distributing and using the COVID-19 vaccine than others. However, vaccine acceptance was affected by social unrest and crime. A study has shown that people who were most concerned about these issues were 3% points less likely to accept the vaccine against COVID-19 [50]. This influence of violence may explain the increase in excess mortality associated with COVID-19, as vaccination is a protective measure against the disease.

Although there was no statistically significant association between “Political Stability and Absence of Violence/Terrorism” and excess mortality in 2020 and 2021, there was a significant and direct association in 2022. One study found that politically stable countries reported more deaths, tests, and cases per capita, as well as higher vaccination coverage compared to corrupt countries. This could be because the most corrupt countries face challenges in registering cases and have lower testing rates, as corruption may have affected the availability of resources and inputs [51].

Greater government effectiveness and less “Political Stability and Absence of Violence/Terrorism” before the pandemic were associated with faster initial responses from the government. This may suggest that “Political Stability and Absence of Violence/Terrorism” function differently from other governance dimensions. However, it is not clear how this dimension affects decision-making in times of crisis [52].

Despite political stability, countries responded differently to the pandemic. Politically stable nations conducted more testing and had higher numbers of cases and deaths due to COVID-19 compared to countries with political instability. Countries with worse governance indicators and more corruption were associated with decreased reporting of cases, deaths, and testing [51]. This may be related to the result of the present research, which showed a direct and significant association between “Political Stability and Absence of Violence/Terrorism” and excess mortality in 2022.

The results of the current study suggest that the covariates of vaccination were not linked to increased mortality in 2021 and 2022. A previous study indicated that countries that achieved more efficient vaccination rates in the initial phase of the pandemic had a reduction in the number of people infected [53]. The difference between these results may be due to the type of outcome analyzed by the studies, which was excess mortality versus the number of people infected. Additionally, vaccination efficiency may be linked to other factors such as the logistical capacity to store and distribute vaccines, and proper communication of the importance of vaccination to the target population.

Surprisingly, countries that made vaccination available to the entire population later had a lower rate of accumulated infection compared to countries that made it available earlier. It was expected that the sooner universal availability of vaccines was achieved, the lower the infection rate would be. The period considered for the accumulated infection rate was from January 1, 2020, to September 30, 2021. In 2020, none of the countries had achieved the availability of vaccines for the entire population, which may have influenced the results. Countries that achieved universal availability of vaccines from January to June 2021 may have had a higher accumulated infection rate in 2020.

A study assessing eight countries, selected based on the criteria of vaccine doses (60 doses per 100 people) and a population of over one million individuals, matched the results of the present research, showing that infection rates in all countries were reduced after vaccination [54].

Our findings showed that the variables related to vaccination were significant and inversely proportional. This means that both the “Vaccination Policies” variable in 2022 and the “Administration of first dose” variable in 2021 were inversely correlated with governance indicators. Countries that adopted vaccination policies or administered the first dose later had lower governance indicators. However, this relationship between governance and vaccination is influenced by several factors such as distribution capacity and hesitation to receive. The availability of vaccines to certain groups, such as frontline healthcare workers, the vulnerable, and the elderly, and achieving universal availability are related to vaccination policies. Moreover, throughout the pandemic, countries with low levels of governance, which initially had low levels of vaccination, achieved approximately half the doses per 100 people of countries with better governance [55]. The present study did not consider the factors related to the storage, transport, and distribution of vaccines as possible confounding factors, which may have influenced the results.

Another study analyzing data from 204 countries showed that good governance was associated with earlier administration of the first dose by 9.1 days. Government effectiveness followed by political stability were the indicators with the largest association [56]. The findings of the present study indicate that while the other vaccination indicators were positively associated with good governance, the “Vaccination Policies” indicator showed a significant and inverse correlation with the governance indicators. The inverse association of the vaccination policies indicator with governance was unexpected, given that this indicator reflects a country’s ability to provide universal access to the COVID-19 vaccine, especially for vulnerable groups such as the elderly, frontline workers, and clinically vulnerable individuals and to the entire population. It is important to investigate further the relationship between governance and vaccine availability, as this can help us prepare better for future pandemics. Our findings corroborate a previous study including 172 countries showing that good governance is associated with higher rates of vaccination against COVID-19 [57]. However, different indicators weighed differently in that study i.e., “Voice and Accountability,“ (22%) “Political Stability and Absence of Violence/Terrorism” (19%) and “Regulatory Quality” (16%) were the indicators that weighed most in vaccination performance, approximately three months after the first vaccine against COVID-19. In our study, “Government Effectiveness” had a stronger correlation with the governance indicators while “Voice and Accountability” had a weaker correlation.

The governance indicators that were associated with lower excess mortality due to COVID-19 were those related to the quality of public services provided, the formulation and implementation of solid policies and regulations, and trust and respect for society’s rules. So, improving and strengthening these aspects can help us face future pandemics better. Controlling corruption can also improve public trust in politicians and the government’s credibility [58, 59]. In other words, strengthening national governance can improve our level of preparation and response to future pandemics.

Our study has some limitations that should be acknowledged. The cross-sectional nature of the study makes it difficult to draw any causal conclusions. The confounding factors considered in this research needed to be limited to a manageable number and do not pretend to represent the hole complexity of the topic. It has to be emphasized that our analysis did not take into account relevant socioeconomic indicators and cultural [60], social and societal values. The research results may have been impacted by missing data and temporal variation in COVID-19 outcomes. It is also important to note that the correlation coefficient analysis does not imply a cause-and-effect analysis.

## Conclusion

The “Control of Corruption, Government Effectiveness, Regulatory Quality, and Rule of Law” component was associated with lower excess mortality rates due to COVID-19. However, the dimensions of this indicator, such as the “Voice and Accountability” component and the “Political Stability and Absence of Violence/Terrorism” component, have differently influenced the performance of countries in controlling this excess mortality.

Investing in good governance can be a powerful strategy for countries to prepare for future pandemics. Our results have shown that countries with better governance indicators had better vaccination indicators, including starting vaccination against COVID-19 earlier than other countries. However, even though good governance is important for pandemic preparedness, it may not be enough to effectively address a pandemic. Studies have shown that governance did not have an impact on reducing COVID-19 deaths.

Moreover, it has to be stressed that national indicators are not always representative of regional or local circumstances. Therefore, future studies should investigate how regional disparities in different countries influenced government actions and national governance during the pandemic. Furthermore, social, economic, and cultural differences between countries should be considered as potential confounding factors when analyzing the effect of national governance on the pandemic.

Lastly, it is crucial to investigate the relationship between governance and other indicators, such as testing capacity, contact tracing, and epidemiological surveillance, and the association of excess mortality due to COVID-19 and other factors, such as social cohesion and risk communication.

### Electronic supplementary material

Below is the link to the electronic supplementary material.

**Supplementary Material 1**: The complete list of 213 countries

**Supplementary Material 2**: Variables of interest, source, description and year

**Supplementary Material 3**: The methodological flow

## Data Availability

An open-source online data repository hosted at primary databases.
