# Supplementary material for: National governance and excess mortality due to COVID-19 in 213 countries: a retrospective analysis and perspectives on future pandemics
Source: Global Health. 2023 Oct 31;19:80. doi: 10.1186/s12992-023-00982-1 (PMC10619274; doi:10.1186/s12992-023-00982-1)
Supplement: Supplementary file 2 — Supplementary Material 2: Variables of interest, source, description and year [file 12992_2023_982_MOESM2_ESM.docx]

| **Supplementary file 2: Variables of interest, source, description and year** | | | |
| --- | --- | --- | --- |
| **Variable** | **Source** | **Description** | **Year** |
| Voice and accountability | World Bank | *“Voice and accountability captures perceptions of the extent to which a country's citizens are able to participate in selecting their government, as well as freedom of expression, freedom of association, and a free media. This table lists the individual variables from each data source used to construct this measure in the Worldwide Governance Indicators. Estimate of governance (ranges from approximately -2.5 (weak) to 2.5 (strong) governance performance)* | 2020 |
| Political Stability No Violence | World Bank | *Political Stability and Absence of Violence/Terrorism measures perceptions of the likelihood of political instability and/or politically motivated violence, including terrorism. Estimate of governance (ranges from approximately -2.5 (weak) to 2.5 (strong) governance performance)* | 2020 |
| Government Effectiveness | World Bank | *Government effectiveness captures perceptions of the quality of public services, the quality of the civil service and the degree of its independence from political pressures, the quality of policy formulation and implementation, and the credibility of the government's commitment to such policies. Estimate of governance (ranges from approximately -2.5 (weak) to 2.5 (strong) governance performance)* | 2020 |
| Regulatory quality | World Bank | *Regulatory quality captures perceptions of the ability of the government to formulate and implement sound policies and regulations that permit and promote private sector development. This table lists the individual variables from each data source used to construct this measure in the Worldwide Governance Indicators. Estimate of governance (ranges from approximately -2.5 (weak) to 2.5 (strong) governance performance)* | 2020 |
| Rule of law | World Bank | *Rule of law captures perceptions of the extent to which agents have confidence in and abide by the rules of society, and in particular the quality of contract enforcement, property rights, the police, and the courts, as well as the likelihood of crime and violence. Estimate of governance (ranges from approximately -2.5 (weak) to 2.5 (strong) governance performance)* | 2020 |
| Control of corruption | World Bank | *Control of corruption captures perceptions of the extent to which public power is exercised for private gain, including both petty and grand forms of corruption, as well as "capture" of the state by elites and private interests. This table lists the individual variables from each data source used to construct this measure in the Worldwide Governance Indicators. Estimate of governance (ranges from approximately -2.5 (weak) to 2.5 (strong) governance performance)* | 2020 |
|  |  |  |  |
| infection-fatality ratio |  | *The IFRs were calculated by applying a 9-day lag to our daily infections to account for the delay between infection and death, calculating the sum of infections and deaths, and then dividing the cumulative deaths over the cumulative lagged infections. For this research, Adjusted IFR per 1000 infections will be used. IFR has been adjusted to population density, gross domestic product (GDP), altitude, and seasonality— factors that might increase transmission—and age, age-standardized chronic obstructive pulmonary disease prevalence, and age-standardized cancer prevalence—factors that might increase morbidity or mortality from infection—and previous exposure to coronaviruses, a factor that might influence both subsequent* *transmission probability and mortality outcomes. These factors are known or considered influence IFR (8).* | 2021 |
| Cumulative infection rate |  | *cumulative infections were calculated by summing up the total estimated daily infections for each national or subnational location over the entire time period (and also for the shorter time period, Jan 1, 2020, to Oct 15, 2020), and were divided by the 2019 estimated population in each location to get the cumulative infections per capita. Adjusted infections per 1000 people will be used. This rate has been adjusted to seasonality, altitude, GDP per capita, population density, previous betacoronavirus exposure (8).* | 2021 |
| Cumulative_estimated_daily_excess_deaths_per_100k | The Economist | *Excess mortality is defined as deaths from all causes during a period, after accounting for expected deaths.14 It estimates how many more people died during the COVID-19 pandemic than would be expected under usual conditions. Excess mortality is a more comprehensive assessment of the pandemic toll than confirmed COVID-19 deaths, as it also captures incorrectly diagnosed or reported deaths and indirect mortality resulting from overburdened healthcare systems or exacerbated poverty. Greater values of excess mortality per 100 000 individuals indicates poor pandemic response.* | 2020; 2021 e 2022 |
| People fully vaccinated (cumulative) | Our World in Data - Oxford Martin School | *Total number of people who received all doses prescribed by the initial vaccination protocol* | Last update (February 15th) |
| Delivered population | United Nations | *Delivered vaccines includes vaccines that have been made available in the country (% of population)* | Last update (February 15th) |
| Vaccination policy | Oxford COVID-19 Government Response  Tracker) | *Countries are grouped into six categories: i) No availability; ii) Availability for ONE of following: key workers/ clinically vulnerable groups / elderly groups; iii) Availability for TWO of following: key workers/ clinically vulnerable groups / elderly groups; iv) Availability for ALL of following: key workers/ clinically vulnerable groups / elderly groups; v) Availability for all three plus partial additional availability (select broad groups/ages); vi) Universal availability.* | - |
| Administration of the  first dose in the country | Our World in Data | *First vaccine date at the country level* | - |
| The total number of vaccination doses administered per 100 people at the country level (cumulative) | Our World in Data | *Number of vaccine doses administered per 100 people within a given population. All doses, including boosters, are counted individually”.* | Last update (February 15th) |
