# Supplementary material for: National governance and excess mortality due to COVID-19 in 213 countries: a retrospective analysis and perspectives on future pandemics
Source: Global Health. 2023 Oct 31;19:80. doi: 10.1186/s12992-023-00982-1 (PMC10619274; doi:10.1186/s12992-023-00982-1)
Supplement: Supplementary file 3 — Supplementary Material 3: The methodological flow [file 12992_2023_982_MOESM3_ESM.docx]

Supplementary file 3: Methodological flow
